# Nanotherapeutics for Treatment of Pulmonary Arterial Hypertension

**DOI:** 10.3389/fphys.2018.00890

**Published:** 2018-07-13

**Authors:** Victor Segura-Ibarra, Suhong Wu, Nida Hassan, Jose A. Moran-Guerrero, Mauro Ferrari, Ashrith Guha, Harry Karmouty-Quintana, Elvin Blanco

**Affiliations:** ^1^Department of Nanomedicine, Houston Methodist Research Institute, Houston, TX, United States; ^2^Escuela de Ingeniería y Ciencias, Tecnológico de Monterrey, Monterrey, Mexico; ^3^McGovern Medical School, The University of Texas Health Science Center at Houston, Houston, TX, United States; ^4^Department of Medicine, Weill Cornell Medicine, New York, NY, United States; ^5^Department of Cardiology, Houston Methodist DeBakey Heart and Vascular Center, Houston Methodist Hospital, Houston, TX, United States; ^6^Houston Methodist J.C. Walter Jr. Transplant Center, Houston Methodist Hospital, Houston, TX, United States; ^7^Department of Biochemistry and Molecular Biology, McGovern Medical School, The University of Texas Health Science Center at Houston, Houston, TX, United States

**Keywords:** pulmonary arterial hypertension, chronic lung disease, nanomedicine, nanoparticles, drug delivery

## Abstract

Pulmonary arterial hypertension (PAH) is a devastating and fatal chronic lung disease. While current pharmacotherapies have improved patient quality of life, PAH drugs suffer from limitations in the form of short-term pharmacokinetics, instability, and poor organ specificity. Traditionally, nanotechnology-based delivery strategies have proven advantageous at increasing both circulation lifetimes of chemotherapeutics and accumulation in tumors due to enhanced permeability through fenestrated vasculature. Importantly, increased nanoparticle (NP) accumulation in diseased tissues has been observed pre-clinically in pathologies characterized by endothelial dysfunction and remodeled vasculature, including myocardial infarction and heart failure. Recently, this phenomenon has also been observed in preclinical models of PAH, leading to the exploration of NP-based drug delivery as a therapeutic modality in PAH. Herein, we discussed the advantages of NPs for efficacious treatment of PAH, including heightened therapeutic delivery to diseased lungs for increased drug bioavailability, as well as highlighted innovative nanotherapeutic approaches for PAH.

## Introduction

Pulmonary arterial hypertension (PAH) is a progressive and fatal disease arising from restricted blood flow through pulmonary arterial circulation. Defined as having mean pulmonary artery pressures (mPAP) greater than 25 mm Hg (Pauwaa et al., 2011), the increased flow resistance in PAH causes an overload in the right ventricle (RV), leading to hypertrophy, hyperplasia, and fibrosis (Ryan and Archer, 2014). These ultimately lead to right heart failure, the major cause of death in PAH patients (Shah, 2012). PAH pertains to the Group I subset of PH, which encompasses idiopathic and heritable disease affecting pulmonary vasculature (Collum et al., 2017). Pathophysiologically, PAH is characterized by remodeling of the pulmonary vasculature that leads to vessel occlusion, muscularization of previously non-muscular vessels, and formation of complex vascular lesions (Stenmark et al., 2009), with pulmonary arteriole smooth muscle cells (PASMCs) and endothelial cells (PAECs) lying at the crux of these processes (Morrell et al., 2009).

Pulmonary arterial hypertension drug therapies have traditionally relied on regulation of vascular tone (Sahni et al., 2016), principally targeting the prostacyclin (PGI_2_), endothelin (ET), and nitric oxide signaling pathways (Lang and Gaine, 2015). While pharmacotherapies have resulted in improvements in hemodynamics and quality of life (Lau et al., 2017), they are not without considerable shortcomings, including short drug half-lives and instability (Delcroix and Howard, 2015), as well as adverse side effects (Galie et al., 2009). Moreover, despite combination drug regimens, PAH undoubtedly progresses despite pharmacotherapy. Thus, there are currently no curative treatments available for PAH patients save for lung transplantation (Gottlieb, 2013), highlighting the pressing need to develop innovative treatments that can attenuate or even reverse vascular remodeling.

Nanotechnology-based drug delivery platforms prove effective vectors for packaging of drug and genetic material (Ferrari, 2005). Nanoparticles (NPs) are defined as possessing diameters between 0.1 and 100 nm, which can be composed of either naturally occurring or synthetic, man-made materials (Riehemann et al., 2009). These nanoconstructs can be precisely designed with regards to size and geometry, with versatile chemistry enabling tailorability of properties such as enhanced cellular entry and controlled release (Blanco et al., 2015). NP platforms prolong circulation lifetimes of drugs when administered intravenously (IV), proving pharmacokinetically advantageous when compared to conventional drug formulations (Blanco et al., 2011). Importantly, the myriad of pathophysiological alterations involved in PAH progression, particularly endothelial injury, provides a potential avenue for systemically administered nanotherapies in PAH. NP-based drug delivery has been extensively used in cancer primarily because of the ability of long-circulating NPs to accumulate passively in tumors by extravasating through leaky vasculature (Maeda et al., 2013). This phenomenon is commonly referred to as the enhanced permeability and retention (EPR) effect. Herein, we will discuss conventional pharmacotherapies in PAH. We will also describe the established NP platforms commonly used for drug delivery, and highlight the role that vascular remodeling in PAH can play in enhancing accumulation in lungs. Lastly, we will showcase several nanotherapeutic strategies that prove promising for the treatment of PAH.

## Conventional Drug Therapy in PAH

### Prostacyclin Agonists

Produced in vascular endothelial cells, the arachidonic acid metabolite PGI_2_ plays an important role in vasodilation, and inhibits smooth muscle cell (SMC) proliferation and platelet aggregation (Del Pozo et al., 2017). By binding and activating the PGI_2_ (IP) receptor on SMCs, PGI_2_ activation increases cyclic adenosine monophosphate (cAMP) levels, which in turn results in vasodilation (Ricciotti and FitzGerald, 2011). In PAH, endogenous PGI_2_ levels are decreased (Tuder et al., 1999), making PGI_2_ and prostaglandin analogs attractive therapeutic options for treatment. Prostanoids have been used clinically over the past three decades for PAH therapy, with the synthetic PGI_2_, epoprostenol sodium (Flolan^®^), being the first pharmacological agent to gain FDA approval for the treatment of PAH (Safdar, 2011), based on improvements in exercise capacity and hemodynamics in patients (Barst et al., 1996).

### Endothelin Receptor Antagonists

Produced by endothelial cells, endothelin-1 (ET-1) promotes SMC vasoconstriction, proliferation, migration, and survival. ET-1 also promotes collagen synthesis by fibroblasts (Rosano et al., 2013). Binding of ET-1 to endothelin receptors (ET_A_ and ET_B_) on SMCs activates phospholipase C, which in turn increases intracellular calcium, resulting in sustained vasoconstriction (Seo et al., 1994). Patients diagnosed with PAH have increased activation of ET-1 in both plasma and lung tissues (Galié et al., 2004) and elevated plasma levels of ET-1 can be correlated with severity of disease and prognosis (McLaughlin et al., 2009), leading to the exploration of various compounds capable of blocking either ET_A_ or ET_A_ and ET_B_ receptors. Three orally administered ET receptor antagonists (ERAs), ambrisentan (Letairis^®^, an ET_A_ receptor inhibitor), bosentan (Tracleer^®^, a dual ET_A_ and ET_B_ receptor inhibitor), and macitentan (Opsumit^®^, a dual ET_A_ and ET_B_ receptor inhibitor), have been clinically approved by the FDA based on randomized clinical trials where increases in 6-min walk distance (6MWD), improved hemodynamic parameters, and overall quality of life were observed (Raja, 2010).

### Nitric Oxide Promoters

Nitric oxide (NO) is a product of endothelial cells and a potent vasodilator. By binding to and subsequent activation of soluble guanylate cyclase (sGC), NO increases levels of cyclic guanosine monophosphate (cGMP) (Russwurm and Koesling, 2004), resulting in reduced intracellular calcium levels and SMC relaxation (Carvajal et al., 2000). NO has also been shown to inhibit SMC proliferation and platelet activation (Tonelli et al., 2013). Levels of NO and NO-products in lungs and bronchoalveolar lavage fluid (BALF) of PAH patients have been shown to be significantly lower compared to control subjects (Kaneko et al., 1998). Therapies targeting the NO pathway in PAH consist of sGC agonists and phosphodiesterase type 5 (PDE5) inhibitors. While NO signaling in PAH patients is aberrant, sGC is expressed in PASMCs of PAH patients (Schermuly et al., 2008), making sGC stimulators attractive agents for increasing cGMP levels in these patients. One such oral sGC agonist, riociguat (Adempas^®^), was the first drug approved targeting the NO pathway for the treatment of PAH, and activates sGC directly despite the absence of NO (Klinger and Kadowitz, 2017). Findings also demonstrate that PDE5 is overexpressed in PASMCs of PAH patients (Murray et al., 2002). PDE5 inhibitors function by hindering the degradation of cGMP (Giovannoni et al., 2010). Administered orally, PDE5 inhibitors currently approved for the treatment of PAH are sildenafil (Viagra^®^) and tadalafil (Cialis^®^). sGC stimulators and PDE5 inhibitors have led to improved 6MWD in patients, as well as lessened time to clinical worsening (Humbert et al., 2014).

### Pitfalls of Conventional Pharmacotherapies

Pharmacotherapies in PAH have improved patient hemodynamics and quality of life, but are not without significant shortcomings. Chief among these are drug half-life, stability, and formulation limitations, resulting in deleterious side effects. As an example, epoprostenol has a short half-life of 3–5 min, and instability at low pH values (Mubarak, 2010). As a result, the drug must be continuously infused IV by means of an implanted catheter and infusion pump, and the drug must be constantly maintained under refrigeration and prepared daily. Consequently, patients are at risk of infections, sepsis, and thrombosis (McLaughlin and Palevsky, 2013). Moreover, permanently implanted catheters may malfunction (Ruan et al., 2010). In the case of drugs such as PDE5 inhibitors, a high and continuous dosage is required to achieve beneficial effects, necessitating oral administration of 80 mg up to 3 times a day (Galie et al., 2005).

An additional pitfall is the non-specific distribution of pharmacotherapies, resulting in adverse systemic side effects. Prostanoid therapy is associated with flushing, headaches, and gastrointestinal symptoms, such as nausea and vomiting (Lang and Gaine, 2015). Traditional ET inhibitors result in peripheral edema, anemia, and hepatotoxicity (Aversa et al., 2015). And while the precise mechanism of liver toxicity has not been fully established, abnormal liver function is an indication for treatment discontinuation (McGoon et al., 2006). Lastly, targeting the NO pathway by either PDE5 inhibitors or sGC stimulators causes side effects such as headache, dyspepsia, peripheral edema, nausea, and dizziness (Ishikura et al., 2000; Ghofrani et al., 2013), in addition to retinal vascular disease and myocardial infarction (Duarte et al., 2013).

Novel drug formulations address limitations related to formulation and delivery. As an example, epoprostenol AS (Veletri^®^), contains arginine and sucrose, and can be stable at room temperature for up to 72 h depending on the concentration of the solution (Sitbon and Vonk Noordegraaf, 2017). More stable prostanoids such as inhaled iloprost (Ventavis^®^) showed improvements in exercise capacity and beneficial hemodynamic effects (LeVarge, 2015). Recently, a non-prostanoid PGI_2_ receptor analog, selexipag (Uptravi^®^) was developed and approved for oral administration in PAH (Duggan et al., 2017). In the case of ERAs, the aforementioned macitentan reduced morbidity and mortality in PAH patients (Sitbon et al., 2014), lowering the incidence of liver toxicity. Despite these improvements, strategies capable of increasing the bioavailability of PAH pharmacotherapies in the lung have the potential to improve patient outcomes and reduce systemic adverse events.

## NP Platforms for Drug and Gene Delivery

### Liposomes

**FIGURE 1:**
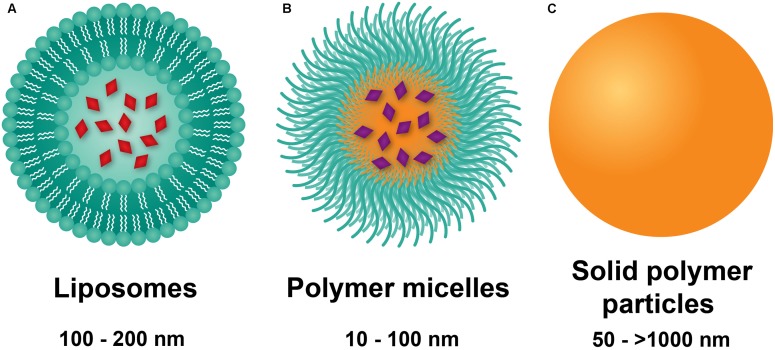
Nanoparticle platforms explored in PAH drug and gene delivery. **(A)** Liposomes are comprised of a lipid bilayer, with an aqueous core ideal for encapsulation of water-soluble drugs (red). **(B)** Polymer micelles are comprised of amphiphilic block copolymers that self-assemble in water to form a hydrophilic corona and a hydrophobic core for encapsulation of lipophilic drugs (violet). **(C)** Solid polymer particles have drug dissolved or embedded within a polymer matrix.

Liposomes are composed of phospholipids with polar heads and hydrophobic tails, forming bilayered constructs with an aqueous core, typically on the order of 100 nm in size (**Figure 1A**) (Pattni et al., 2015). The aqueous compartment is ideal for accommodation of water-soluble drugs. Hydrophobic drugs can be incorporated within the bi-phospholipid membrane, albeit at the risk of membrane destabilization (Liu et al., 2006). Functionalization of liposomes with polyethylene glycol (PEG) on the surface led to significant enhancement of circulation lifetimes, best demonstrated by DOXIL^®^, a PEGylated liposomal formulation of doxorubicin (Hamilton et al., 2002). The increase in circulating half-life was a direct result of incorporating PEG onto the surface of liposomes, with the hydrating layer provided by PEG deterring protein adsorption and NP clearance by the mononuclear phagocyte system (MPS) (Harris and Chess, 2003). Importantly, liposomal doxorubicin was shown to reduce doxorubicin-associated cardiotoxicity compared to the conventional, clinically used formulation of doxorubicin (Berry et al., 1998). These advantages led to DOXIL^®^ being the first NP platform approved by the FDA for the treatment of Kaposi’s sarcoma in 1995 (Barenholz, 2012). Liposomes also prove advantageous for efficient delivery of genetic material through incorporation of cationic lipids such as [1,2-bis(oleoyloxy)-3-(trimethylammonio)propane] (DOTAP) (Zhang et al., 2012). Functionalization of liposomes with the thermoresponsive polymer N-isopropylacrylamide (NIPAAm) can be used to induce membrane disruption at high temperatures, resulting in increased local release of drug at specific sites (Ta and Porter, 2013).

### Polymer Micelles

Polymer micelles are NPs formed from the self-assembly of amphiphilic-block copolymers in aqueous environments (Blanco et al., 2009). The core-shell morphology of polymer micelles consists of a hydrophobic core and a hydrophilic shell (**Figure 1B**), wherein the hydrophilic block of the constituent polymer is typically PEG. On the order of 10–100 nm in diameter, polymer micelles have traditionally been used as delivery vehicles for hydrophobic drugs. Of significant note, the tailorability of polymer chemistries makes micelles highly versatile carriers with a myriad of advantages for drug delivery. Cationic polymers such as polyethylenimine (PEI) (Dai et al., 2011b) or poly(L-lysine) (Christie et al., 2012) can be either grafted onto block copolymers or used as the core-forming block for loading of genetic material. Stimuli-responsive, tailored drug release can also be obtained based on the composition of the core forming polymer block. As an example, Bae et al. (2005) used PEG-b-poly(aspartate) (PEG-PAsp) for pH-sensitive release of doxorubicin by conjugating it to PAsp through a hydrazine linkage. Lastly, targeting moieties including antibodies, aptamers, and peptides fashioned onto polymer micelles can be used for active targeting to diseased tissues and cells (Jhaveri and Torchilin, 2014). As an example, the cyclic(Arg-Gly-Asp-DPhe-Lys) (cRGDfK) peptide has been used for polymer micelle targeting to the a_v_b_3_ integrin found overexpressed on tumor vasculature (Nasongkla et al., 2004; Song et al., 2014). Despite their numerous advantages, polymer micelles are limited by fast release of drug and long-term stability, with strategies such as interlayer-crosslinked cores (Dai et al., 2011a) shown to prevent premature drug release.

### Solid Polymer Particles

Solid polymer particles, typically comprised of the polyester polylactide-co-glycolide (PLGA), have long been employed in controlled drug release applications. These particles are spherical in morphology, can range from the nano- to micro-meter dimensions, and can be used for delivery of water soluble and insoluble drugs (Makadia and Siegel, 2011), with agents dissolved or encapsulated within the polymer matrix (**Figure 1C**; Danhier et al., 2012). PLGA remains the constituent polymer of choice for these NPs due to several advantages. Chief among these is the relative ease of fabrication, as well as the biocompatibility and biodegradability of the PLGA, a material approved by the FDA for a wide range of biomedical applications. In aqueous environments, ester linkages of PLGA undergo hydrolysis, producing the monomers lactic acid and glycolic acid, which are readily metabolized and removed from the body (Acharya and Sahoo, 2011). Moreover, drug release from PLGA NPs occurs through initial diffusion followed by degradation of the polymer matrix, which in turn is affected by crystallinity, composition, molecular weight, and size and shape of the matrix (Makadia and Siegel, 2011). Thus, highly controllable and sustained release profiles can be achieved by employing PLGA copolymers with the more hydrophobic polylactic acid (PLA) than polyglycolic acid (PGA), which give rise to NPs with less water absorption and slower degradation kinetics (Dinarvand et al., 2011). In addition to drugs, PLGA particles can incorporate cationic polymers (e.g., PEI) for delivery of genetic material (Bivas-Benita et al., 2004). PLGA NP drug delivery is limited by rapid initial release of payload due to hydration of the polymer (Kapoor et al., 2015), as well as dose dumping effects at longer timepoints (Khanal et al., 2016). Moreover, peptides and proteins may undergo chemical degradation within polymer matrices (Houchin and Topp, 2008).

### Nanoparticle Size Considerations

The relative size of the different NPs influences *in vivo* fate following intravenous delivery. It is now well known that NPs with diameters < 5 nm are cleared rapidly by the kidneys (Choi et al., 2007). NPs that measure > 100 nm accumulate non-specifically in livers (Braet et al., 2007), those measuring > 200 nm accumulate in the spleen (Chen and Weiss, 1973), and particles >2 μm accumulate in lung capillaries. Resident macrophages of the liver, spleen, and lungs rapidly internalize opsonized NPs in a size-dependent manner. Taken together, smaller sized NPs, measuring 100 nm or less, have been shown to be long circulating following intravenous administration (Blanco et al., 2010).

These size considerations play an important role in the design of nanotherapeutic constructs for purposes of targeting specific tissues. As an example, Xu et al. (2016) used particles with a diameter of 2.5 μm to specifically target breast cancer metastasis in the lung. Long-circulating NPs have a heightened propensity to passively accumulate in tissues with remodeled vasculature by extravasating through submicron sized pores in the endothelium (Hobbs et al., 1998). And while smaller sized NPs are able to extravasate from circulation into these diseased sites, the extent of NP penetration into the tissue depends on the size of the carrier. Cabral et al. (2011) were able to demonstrate that sub-100 nm NPs were able to penetrate into permeable tumors. However, in more fibrotic tumors, only NPs measuring < 50 nm were capable of penetrating into the tissue.

Inhalational delivery of NPs represents an attractive strategy for specifically targeting pulmonary tissues. However, particle size also dictates regional lung deposition after inhalation (Paranjpe and Muller-Goymann, 2014). When administered as a dry powder, large particles in the size range of 1–5 μm deposit in bronchioles and smaller airways, particles in the size range of 0.5–1 μm accumulate in alveolar regions, and smaller NPs (< 0.5 μm) can undergo exhalation.

## Enhanced NP Accumulation in Lungs Undergoing PAH

Nanoparticle platforms such as liposomes and polymer micelles have been extensively explored in chemotherapy. While advantageous at increasing the circulation lifetimes of chemotherapeutics, it was the observation by Maeda et al. (2013) regarding the ability of IV-administered macromolecules to accumulate to a large extent in tumors that led to the excitement of NP-based drug delivery strategies in cancer (Matsumura and Maeda, 1986). Passive targeting of macromolecules and NPs to tumors is owed to the high degree of fenestrations (e.g., openings) present in tumor vasculature (McDonald and Choyke, 2003), a direct result of chaotic and ongoing angiogenic processes in tumors (Fang et al., 2011). This enhanced NP accumulation in tumors, combined with NP persistence due to impaired lymphatic drainage (Banerjee et al., 2011) is known as the EPR effect (Maeda et al., 2013).

While passive accumulation of NPs in disease sites is primarily associated with cancer, vascular permeability is prevalent in other diseases characterized by abnormal angiogenesis and vascular remodeling as a consequence of inflammation (Durymanov et al., 2017). As an example, in rheumatoid arthritis, where a combination of angiogenic and inflammatory processes promote vessel leakiness, several groups have reported passive targeting to the synovium (Metselaar et al., 2004; Anderson et al., 2010). Similarly, formation of new blood vessels in atherosclerotic plaques leads to enhanced NP uptake in these lesions (Chono et al., 2005; Stigliano et al., 2017). Vascular injury stemming from local inflammatory processes and hypoxia is present in diseases such as myocardial infarction and heart failure, resulting in enhanced vascular permeability to the heart. Nagaoka et al. (2015) and Nakano et al. (2016) demonstrated increased NP uptake in myocardial infarct areas following IV administration in a model of ischemia-reperfusion (IR) injury in the heart, mirroring previously published findings (Dvir et al., 2011; Paulis et al., 2012). Our laboratory recently demonstrated enhanced accumulation of micron-sized particles in failing hearts compared to healthy hearts (Ruiz-Esparza et al., 2016). It is important to note that the prevalence of immune-related cells in areas of inflammation can also contribute to increased uptake at these sites through macrophage phagocytosis (Ulbrich and Lamprecht, 2010).

**FIGURE 2:**
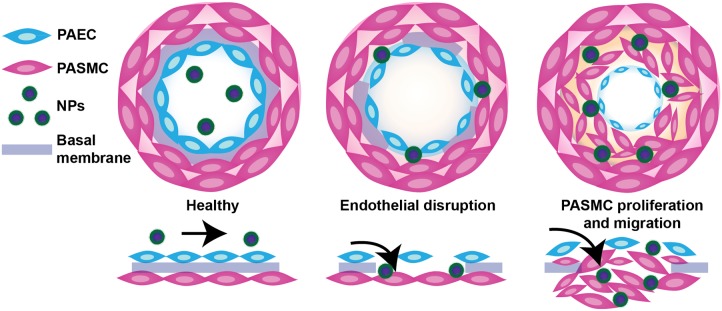
Schematic representation of endothelial dysfunction in PAH and a proposed mechanism of NP extravasation into pulmonary vasculature. While healthy pulmonary vasculature is a semipermeable membrane barrier, the vasculature in lungs undergoing PAH exhibits endothelial dysfunction arising from a chronic inflammatory state and hypoxia, leading to fenestrations (openings) in the endothelium and a hyperpermeable state. Such permeability may be exploited by NPs to passively extravasate and accumulate in lungs undergoing PAH. Figure adapted from Segura-Ibarra et al. (2017), reproduced with permission courtesy of Elsevier.

**FIGURE 3:**
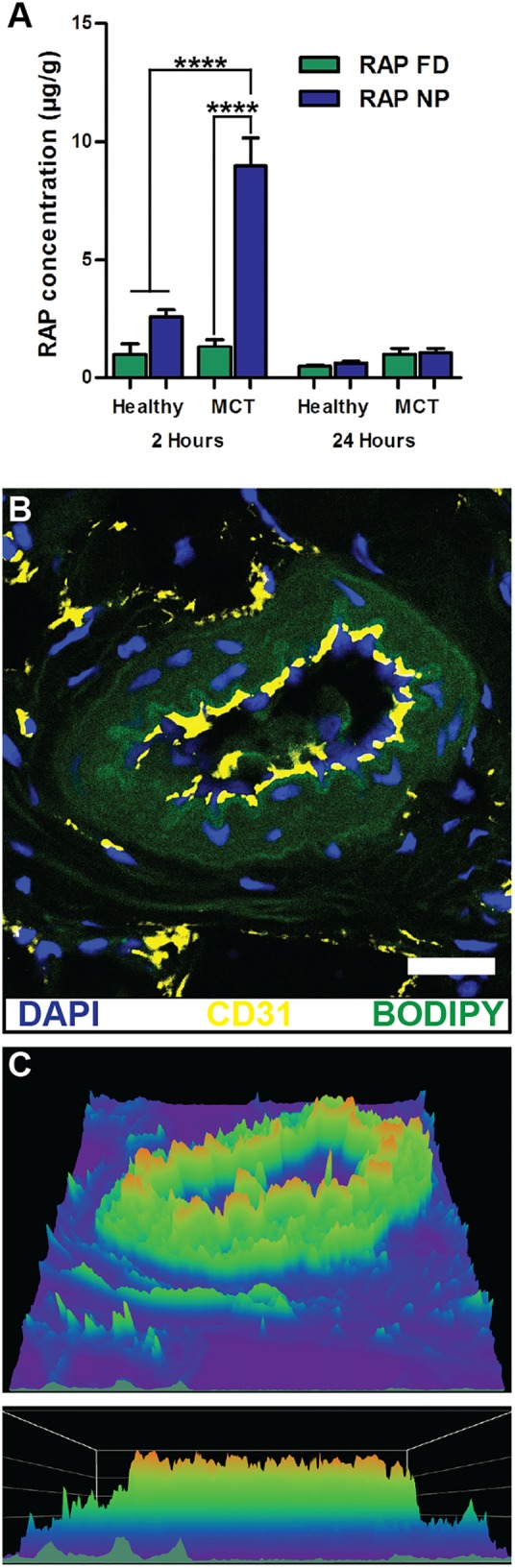
NP accumulation in PAH lung vasculature. **(A)** LC/MS analysis of rapamycin (RAP) concentration in lung tissues 2 and 24 h after a single administration of 15 mg/kg of RAP, either as a free drug formulation (RAP FD) or nanoparticle form (RAP NP) in healthy and MCT-induced model of PH in rats (PH). Results represent mean ± SEM (^∗∗∗∗^*P* < 0.0001). **(B)** Confocal imaging depicting fluorescently loaded NPs in diseased lungs. CD31 positive endothelial cells appear in yellow, NPs are green, while DAPI appears as blue. The scale bar represents 25 μm. **(C)** Surface intensity plot of the image from panel B representing NP signal. Figure adapted from Segura-Ibarra et al. (2017), reproduced with permission courtesy of Elsevier.

Vascular permeability in PAH arises from injurious events such as inflammation and hypoxia, resulting in focal disruptions in endothelial cell basement membranes (McLaughlin and McGoon, 2006; Stenmark et al., 2006; Montani et al., 2014), as well as increased vascular pressure, which leads to fenestrations as a result of greater mechanical and shear stress (**Figure 2**) (Zhou et al., 2016). Moreover, mutations in bone morphogenetic protein receptor 2 (BMPR2), highly prevalent in heritable PAH, have been shown to contribute to increased vascular permeability through dysregulation of the TGF-β signaling pathway (Morrell, 2006). Our laboratory recently demonstrated that vascular permeability in PAH contributes to enhanced NP accumulation in diseased lungs (Segura-Ibarra et al., 2017), agreeing well with previous findings by Ishihara et al. (2015). In a monocrotaline (MCT)-induced model of PAH, poly(ethylene glycol)-*block*-poly(ε-caprolactone) (PEG-PCL) micelles containing rapamycin (RAP) resulted in increased drug accumulation in diseased lungs compared to healthy lungs 2 h after IV administration (**Figure 3A**). Moreover, LC/MS analysis comparing RAP-containing micelles and a free drug formulation of RAP showed a significantly higher increase in RAP accumulation in diseased lungs when packaged within NPs (**Figure 3A**). Upon closer examination of remodeled vasculature using confocal microscopy, heightened accumulation of PEG-PCL NPs was observed within the perivascular region (**Figures 3B,C**).

## Nanotherapeutics in PAH

Conventional pharmacotherapies for PAH treatment suffer from short half-lives, drug instability, and adverse side effects. NP-based strategies for the treatment of PAH offer advantages of improving short-term pharmacokinetics associated with drugs and increased localization of therapy to diseased tissues, in turn decreasing adverse effects. Herein, we highlight nanotherapeutic approaches aimed at delivering clinically approved PAH drugs, as well as nanoplatforms for delivery of novel agents, including genetic material (**Table 1**).

**Table 1:** Nanotherapeutics explored pre-clinically in PAH.

Therapeutic agent	NP formulation	Size	Control	Advantage over control	Model	Reference
Iloprost	Liposomes (various formulations combining POPC, DOTAP, PVP, SA, DPPE-PEG2000, CH)	168–178 nm	Free iloprost	∼1-fold ↑ vasodilation	BALB/c isolated intrapulmonary arteries	Jain et al., 2014
Beraprost	PEG-PLA NP	∼128 nm	Free beraprost	↓ effective dose (20 μg/kg for NP vs. 100 μg/kg for control)	Rat MCT-induced PAH	Ishihara et al., 2015
Beraprost	PLGA NP	280–300 nm	Drug-free vehicle	1.3-fold ↑ survival rate in MCT model, ↓ RV hypertrophy, ↓ RVSP, ↓ muscularized pulmonary arteries in MCT and sugen/hypoxia models	Rat MCT-induced PAH, Rat sugen/ hypoxia-induced PAH	Akagi et al., 2016
NO	Liposomes (EDPPC, DOPC, CH, Ar)	–	NO in Ar saturated mannitol solution	7-fold ↑ NO uptake by VSMC	Cultured VSMC	Huang et al., 2009
NO	Hydrogel-like polymer NP (Methyl silicate, oligochitosan, PVP, PEG)	200–230 nm	Same formulation applied to healthy mice	Concentration-dependent ↑ vasodilation	Mice hypoxia-induced PAH	Mohamed et al., 2016
Pitavastatin	PLGA NP	∼196	Free pitavastatin	↓ RVSP, ↓ arteriolar remodeling, ↓ macrophage infiltration, > 50% ↓ NF-κB positive cells, ↑ survival, ↑ NOS expression	Rat MCT-induced PAH	Chen et al., 2011
Fasudil	Aerosolized Liposomes (DPPC, CH)	∼180 nm	Free fasudil	10-fold ↑ drug half-life, ↑ duration of vasodilation	Rat MCT-induced PAH	Gupta et al., 2013
Fasudil	Liposomes (DPPC, CH, DSPE-PEG, CAR peptide)	206–216 nm	Free fasudil	34-fold ↑ drug half-life in healthy rats; ↓ mPAP (40% reduction for NP vs. 35% for control in MCT model)	Healthy rats, Rat MCT-induced PAH	Nahar et al., 2014
Fasudil, SOD	Liposomes (DPPC, CH, DSPE-PEG-MAL, CAR peptide)	∼150 nm	Fusudil + SOD	↓ mPAP, ↓ arterial medial wall thickness, ↑ vasodilatory effects duration	Rat MCT-induced PAH	Gupta et al., 2017
Ethyl pyruvate	PEG-LG NP	∼286 nm	Free ethyl pyruvate	56% ↓ mPAP, > 50% ↓ arterial medial wall thickness, ∼50% ↓ IL-6, ↓ T N F α, > 50% ↓ ROS, > 6 0 % ↓ HMGB 1	Rat Shunt flow-induced PAH	Liu et al., 2016
Imatinib	PLGA NP	280–300 nm	Drug-free vehicles	∼40% ↓ RVSP, prevented ↑ in RV hypertrophy, ∼50% ↓ small pulmonary vessel muscularization	Rat MCT-induced PAH	Akagi et al., 2015
Rapamycin	PEG-PCL NP	∼17 nm	Free rapamycin	∼50% ↓ Inflammatory cytokines levels, 10% ↓ in weight loss	Rat MCT-induced PAH	Segura-Ibarra et al., 2017
NF-κB decoy oligodeoxy-nucleotide	PEG-PLGA NP	∼44 nm	Free NF-κB decoy	↓ RVSP, ↓ RV hypertrophy, ↓ small pulmonary vessel muscularization, > 50% ↓ inflammatory cytokine mRNA, > 50% ↓ NF-κB positive cells	Rat MCT-induced PAH	Kimura et al., 2009
Anti-sense oligonucleotide against miR-145	Liposomes (Star:Star-mPEG-550)	80–100 nm	Non-silencing oligonucleotide	∼25% ↓ RVSP, ↓ in RV hypertrophy, ↓ arterial medial wall thickness, > 50% ↓ in miR-145 expression	Rat Sugen/ Hypoxia-induced PAH	McLendon et al., 2015


*POPC, 1-palmitoyl-2oleoyl-sn-glycero-3-phosphocholine; DOTAP, 1,2-di-(9Z-octadecenoyl)-3-trimethylammonium-propane; PVP, polyvinylpyrrolidone; SA, stearylamine; DPPE-PEG2000, [methoxy (polyethyleneglycol)-2000]-dipalmitoyl-phosphatidylethanolamine; PEG, polyethylene glycol; PLA, polylactic acid; NP, nanoparticle; MCT, monocrotaline; PAH, pulmonary arterial hypertension; PLGA, poly(lactic-co-glycolic acid); RV, right ventricle; RVSP, right venticular systolic pressure; NO, nitric oxide; EDPPC, 1,2-dipalmitoyl-sn-glycero-3-ethylphosphocholine; DOPC, 1,2-dioleoyl-sn-glycero-3-phosphocholine; CH, cholesterol; Ar, argon; VSMC, vascular smooth muscle cells; SP, systolic pressure; NF-κB, nuclear factor kappa-light-chain-enhancer of activated B cells; NOS, nitric oxide synthase; DPPC, 1,2-dipalmitoyl-sn-glycero-3-phosphocholine; DSPE-PEG-MAL, 1,2-distearoyl-sn-glycero-3-phosphoethanolamineN-[maleimide(polyethylene glycol)-2000]; CAR peptide, peptide with amino acid sequence CARSKNKDC; mPAP, mean pulmonary arterial pressure; PEG-LG, poly(ethylene glycol)-block-lactide/glycolide copolymer; TNFα, tumor necrosis factor alpha; HMGB1, high mobility group box 1 protein; ROS, reactive oxygen species; IL-6, Interleukin 6; PEG-PCL, poly(ethylene glycol)-block-poly(ε-caprolactone); Star, staramine; mPEG, methoxypolyethylene glycol.*

### Prostanoid-Containing NPs

The clinically approved drug inhaled iloprost has an extremely short half-life, requiring at most 12 inhalations per day (Olschewski et al., 2000), largely impacting patient compliance. In hopes of increasing drug bioavailability, Kleemann et al. (2007) developed a liposomal formulation for sustained release of iloprost for aerosolized PAH therapy. Liposomes consisted of di-palmitoyl-phosphatidyl-choline (DPPC), cholesterol to enhance sustained delivery, and poly(ethylene glycol)-di-palmitoyl-phosphatidyl-ethanolamine (PEG-DPPE) to prevent clearance by alveolar macrophages, which would limit their bioavailability. Resulting liposomes ranged in size from 200 to 400 nm, and contained 11 μg iloprost/ml, which would significantly reduce the number of inhalations required.

Jain et al. (2014) fabricated iloprost-containing liposomes with cationic lipids in hopes of increasing drug loading efficiency and examined their efficacy based on changes in vascular tone of pulmonary arteries isolated from mice by means of a wire myograph. NPs averaged 168–178 nm in diameter and had drug loading efficiencies of ∼50%. Pulmonary arteries were constricted by application of the thromboxane analog, U-46619, and treated either with free or liposomal iloprost. Liposomal iloprost resulted in significant enhancement of vasodilation (29% compared to 16% for free iloprost), with a much lower concentration of liposomal iloprost required to bring about efficacies similar to that of free drug.

The oral PGI_2_ analog beraprost has proven vasodilatory and anti-platelet activity, but much like other prostanoids, has a very short half-life (∼1 h) (Barst et al., 2003). In attempts to overcome pharmacokinetic limitations of the drug, Ishihara et al. (2015), who previously formulated NPs containing prostaglandin E1 (PGE1) (Takeda et al., 2009), encapsulated beraprost within poly(ethyleneglycol)-*block*- poly(lactide) (PEG-PLA) micelles and examined their efficacy in an MCT-induced PAH rat model and hypoxia-induced mouse model of PAH. Resulting NPs possessed average diameters of 128 nm and exhibited slow drug release kinetics (∼20% over 1 week). Beraprost NPs showed significantly reduced drug clearance from plasma compared to free beraprost, the former present in circulation at timepoints of 24 h, while the latter was cleared within 6 h. Upon IV administration in an MCT-induced model of PAH in rats, NPs accumulated more in MCT-damaged lungs compared to healthy control lungs, and were found associated with pulmonary peripheral arteries. Importantly, once a week IV administration of beraprost NPs at a dose of 20 μg/kg in an MCT-induced PAH rat model reduced pulmonary arterial remodeling and right ventricular hypertrophy; the efficacy proving similar to that of a daily oral administration of the drug at a much higher dose (100 μg/kg). A similar improvement in pulmonary arterial remodeling was observed in the hypoxia-induced model in mice. This study effectively highlights the advantages afforded by NP-based drug delivery, mainly the need for lower doses and less frequent administrations to achieve similar efficacious responses.

In another study, Akagi et al. (2016) fabricated PLGA NPs containing beraprost and examined the efficacy of the platform in MCT- and Sugen/Hypoxia-induced models of PAH. After a single intratracheal administration of beraprost-containing NPs, RV systolic pressure (RVSP), RV hypertrophy, and the percentage of fully muscularized small pulmonary arteries were significantly reduced compared to disease controls in both PAH models. Moreover, the survival rate increased to 65% following administration of NP-based beraprost, compared to 27.8% in disease controls. Of note, NPs administered intratracheally in the Sugen/Hypoxia-induced model of PAH were found associated with the media of pulmonary arteries and interstitium at timepoints of up to 3 days, whereas no NPs were evident in healthy control lungs.

### NP-Based Targeting of the NO Pathway

Nitric oxide plays an important role in healthy pulmonary physiology, driving SMC relaxation (Perez-Zoghbi et al., 2010), with added anti-inflammatory and proliferative properties (Tonelli et al., 2013). Huang et al. (2009) developed a liposomal formulation of NO consisting of 1,2-dipalmitoyl-sn-glycero-3-ethylphosphocholine (EDPPC), 1,2-dioleoyl-sn-glycero-3-phosphocholine (DOPC), and cholesterol. These liposomes encapsulated 10 μL of NO per mg of lipids and Argon (Ar) was used as an excipient for NO. Upon examination of release kinetics *in vitro*, release of NO from liposomes was slower in the presence of Ar, resulting in a sustained release profile. No significant toxicity was observed *in vitro* in cultured rat vascular smooth muscle cells (VSMCs), and based on a colorimetric NO assay kit, a sevenfold increase in uptake of NO was observed with liposomal NO than NO formulated in Ar saturated mannitol solution. Moreover, liposomes protected NO from microenvironmental scavengers such as hemoglobin. To evaluate *in vivo* efficacy, a balloon injury was induced in the common carotid arteries of rabbits and liposomes containing NO were administered locally. After 2 weeks, a significant decrease in intimal hyperplasia was observed in rabbits treated with liposomal NO compared to vehicle controls (empty liposomes), demonstrating the feasibility of delivery of bio-active gases NPs.

Recently, Mohamed et al. (2016) developed a novel hydrogel-like polymer composite NP formulation for delivery of NO. NPs released NO in a sustained fashion over time, and showed concentration-dependent vasodilation of U-46619-induced preconstricted pulmonary arteries, with a more pronounced effect observed in arteries from hypoxia-induced PAH mice compared to healthy mice.

Beck-Broichsitter et al. (2012) have explored novel spray-drying techniques to fabricate PLGA microparticles for deposition in the lungs and release of sildenafil. Using a vibrational spray drying procedure, resulting microparticles measured 4–8 μm in size and had a high sildenafil encapsulation efficiency of > 90% (Beck-Broichsitter et al., 2017). Moreover, the formulation resulted in a sustained release of sildenafil over time, making these microparticles potentially beneficial for controlled pulmonary drug delivery in PAH and chronic lung diseases.

### Nanotherapeutic Delivery of Novel Agents Targeting PAH

Currently, statins are one of the first-line medications given to patients with elevated cholesterol levels to prevent cardiovascular disease. The mechanism of action involves inhibiting the rate-limiting step of cholesterol biosynthesis by competitive inhibition of HMG-CoA reductase (Istvan, 2003). Statins also improve endothelial function (Beckman and Creager, 2006), displaying anti-tumoral (Crescencio et al., 2009), anti-proliferative (Kamigaki et al., 2011), and anti-inflammatory (Ridker et al., 1999; Lefer, 2002) effects.

**FIGURE 4:**
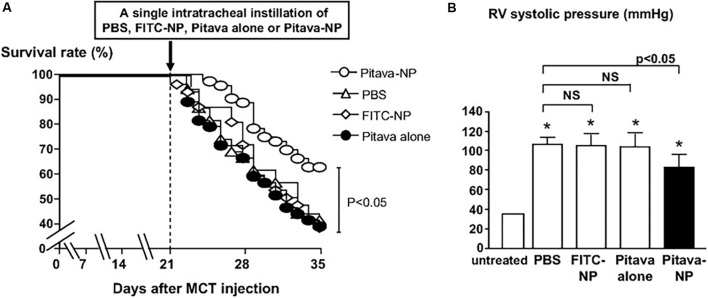
Effects on survival and RV systolic pressures following pitavastatin-NP delivery. **(A)** Kaplan–Meier curve depicting survival of MCT-induced PAH rats following a single intratracheal administration of Pitavastatin-NP compared to controls consisting of intratracheal delivery of PBS, free pitavastatin, and fluorescein isothiocyanate (FITC)-NPs. **(B)** Effects of pitavastatin-NPs on RV systolic pressure (expressed as mmHg). ^∗^*P* < 0.01 vs. untreated control. Figure adapted from Chen et al. (2011), reproduced with permission courtesy of Wolters Kluwer Health, Inc.

Given that inflammation, endothelial injury, and cellular proliferation play a crucial role in PAH progression, Chen et al. (2011) explored the use of statin nanotherapeutics for treatment of PAH. The anti-proliferative effects of different statins (pravastatin, losuvastatin, simvastatin, atorvastatin, fluvastatin, and pitavastatin) were evaluated in human PASMCs, and pitavastatin was selected for PLGA NP encapsulation based on its potent effects. Distribution of PLGA NPs following intratracheal instillation were examined, and FITC-containing NPs were found in lungs of rats undergoing MCT-induced PAH 3 days after administration, specifically in small arteries, bronchi, alveoli, and alveolar macrophages. Of significant note, FITC was detected in lungs at timepoints of up to 14 days after a single administration. A single administration of pitavastatin-containing NPs was performed at the time of PAH induction of rats, and 21 days after administration, right ventricular catheterization revealed a significant decrease in RV systolic pressure compared to rats treated with free pitavastatin or vehicle controls. A significant decrease in systolic pressure in pulmonary arterioles was also observed. Of note, lower levels of macrophages and monocytes were found in rats treated with pitavastatin-containing NPs. Moreover, compared to free pitavastatin, the NP formulation resulted in a > 50% decrease of cells positive for nuclear factor kappa-light-chain-enhancer of activated B (NF-κB), which plays an important role in cell proliferation and survival (Hoesel and Schmid, 2013). The NP formulation increased expression of endothelial NO synthase (eNOS), which can potentially promote endothelial healing. Following NP administration in rats 21 days after MCT induction of PAH, pitavastatin-containing NPs significantly increased survival by 64% compared to control groups (**Figure 4A**) and significantly decreased RVSP compared to disease controls (**Figure 4B**). It is important to note that a Phase I clinical trial involving pitavastatin PLGA NP-based delivery for PAH has recently been completed (Nakamura et al., 2017).

Activation of the Ras homolog gene family, member A (RhoA) GTPase and it downstream effector, the Rho-associated kinase (ROCK), have been implicated in several processes driving PAH pathogenesis, including SMC vasoconstriction and proliferation, and endothelial cell contraction (Oka et al., 2008). Thus, inhibitors of RhoA/ROCK signaling such as Fasudil can potentially prove efficacious in the treatment of PAH. However, Fasudil has a short half-life of ∼45 min (Shibuya et al., 2005). In light of these limitations, Gupta et al. (2013) developed a liposomal formulation of fasudil for purposes of aerosolized delivery to lungs undergoing PAH. Resulting liposomes measured ∼180 nm following nebulization, had loading efficiencies > 60%, and released ∼70% of the drug over the course of 35 h. Pulmonary delivery of liposomes via intratracheal administration increased the half-life by more than 10-fold, as well as the bioavailability of the drug, compared to a free drug formulation administered IV. Upon efficacy examination in an MCT-induced PAH model in rats, an intratracheally administered liposomal formulation of fasudil was compared to a free formulation of fasudil administered intratracheally and by IV. Liposomal fasudil resulted in an increase in the duration of vasodilatory effects compared to controls, with a maximal reduction in mPAP of ∼40%.

In an attempt to enhance site-specific accumulation of NPs to the lungs, Nahar et al. (2014) subsequently developed fasudil liposomes with the cyclic peptide CARSKNKDC, which binds to cell surface heparan sulfate found overexpressed in pulmonary vasculature in PAH. Liposomes were in the range of 206–216 nm and had a sustained release of fasudil over the course of 120 h. Peptide-coated liposomes resulted in ∼34-fold increase in half-life of the drug compared to an IV-administered formulation of free drug. As a result, the mPAP in an MCT-induced model and a Sugen/Hypoxia model of PAH in rats was greatly reduced compared to controls. In a recent study, Gupta et al. (2017) incorporated superoxide dismutase (SOD) into their peptide-targeted fasudil liposomes, with the hypothesis that inclusion of a reactive oxygen species (ROS) scavenger would further enhance efficacy, given the role that increased ROS levels play in vascular remodeling in PAH. In an MCT-model of PAH, wherein the liposomal formulation was administered every 72 h for 21 days, the duration of vasodilatory effects was significantly increased in rats receiving targeted liposomes containing both fasudil and SOD compared to free drug controls. In a Sugen/Hypoxia model of PAH, mPAP, RV hypertrophy, fractions of occluded blood vessels, and arterial medial wall thickness were all reduced in rats receiving targeted liposomes containing both fasudil and SOD compared to free drug controls.

Liu et al. (2016) also examined the potential of ROS scavenging nanotherapeutics for the treatment of PAH. In their study, ethyl pyruvate, a derivative of pyruvic acid and an inhibitor of nuclear protein HMGB1, which in turn activates pro-inflammatory cytokines, was incorporated within poly(ethylene glycol)-*block*-lactide/glycolide (PEG-LG) NPs and examined their efficacy in a hyperkinetic model of PAH induced by shunt flow. At a timepoint of 24 h after intratracheal instillation, NPs were evident in lungs, predominantly in bronchi, alveoli, alveolar macrophages, and small arteries, with evidence of NPs present up to timepoints of 7 days. Following weekly administration of ethyl pyruvate NPs immediately after model induction for a time period of 12 weeks, medial wall thickness index (TI) and medial wall area index (AI) of small pulmonary arteries was significantly reduced by >50% compared to free ethyl pyruvate controls. Moreover, IL-6 and TNFα levels were significantly reduced (∼50%), as were levels of HMGB1 and ROS by more than 50 and 60%, respectively.

PASMC abnormal proliferation is vital to pathogenesis of PAH, with platelet–derived growth factor (PDGF) stimulation resulting in increased growth rate of PASMCs (Ikeda et al., 2010). Akagi et al. (2015) incorporated the PDGF-receptor tyrosine kinase inhibitor imatinib in PLGA NPs and examined their efficacy in an MCT-induced model of PAH. Imatinib is used for the treatment of chronic myelogenous leukemia (CML) and acute lymphocytic leukemia (ALL), and has resulted in 10-year progression-free survivals of 82% in CML (Kalmanti et al., 2015). It is important to note that a limitation of imatinib is patient resistance due to BCR-ABL1 amplification and multidrug-resistant P-glycoprotein (MDR-1) overexpression (Milojkovic and Apperley, 2009). Following a single intratracheal administration immediately after model induction, imatinib-containing NPs significantly reduced RV systolic pressure (∼40% reduction) and RV hypertrophy, as well as muscularization of pulmonary small vessels (∼50% reduction) compared to vehicle controls.

**FIGURE 5:**
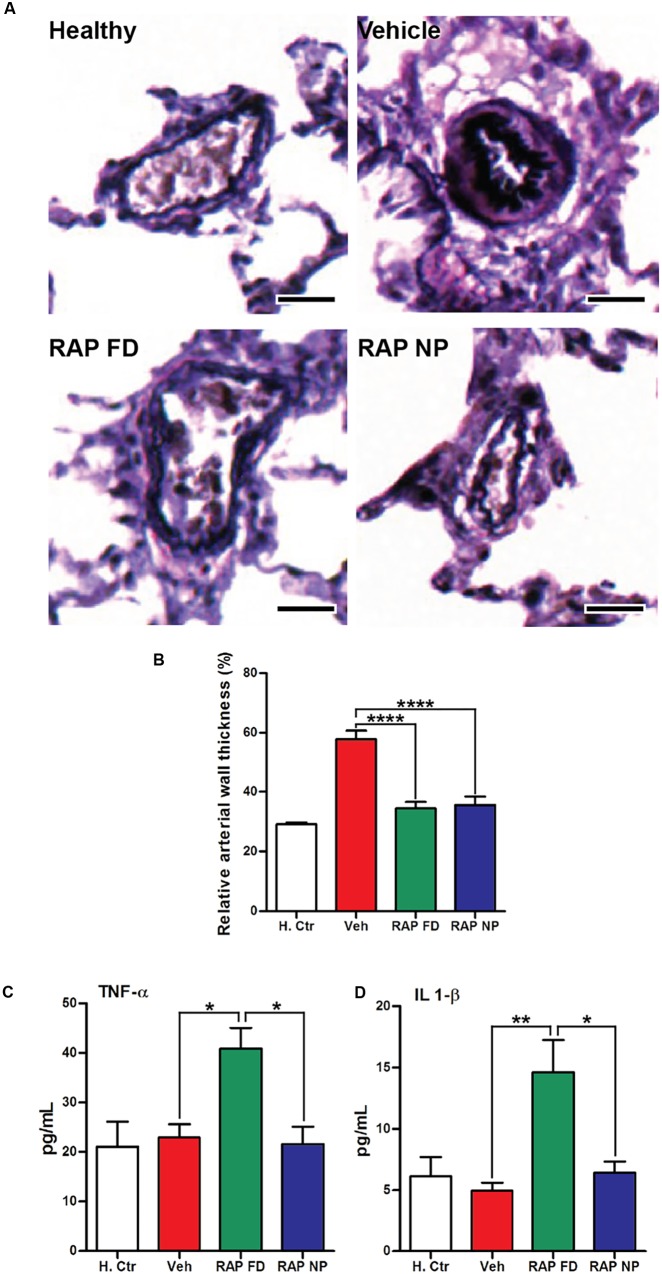
Rapamycin NPs prevented pulmonary arteriole hypertrophy in PAH and did not lead to an increase in inflammatory cytokines. **(A)** Verhoeff–Van Gieson (VVG) stain of pulmonary arterioles from MCT-induced model of PAH in rats treated with free rapamycin (RAP FD), NP vehicle (Vehicle), and RAP NPs. Scale bars represent 50 μm. **(B)** Quantification of the relative wall thickness among treated groups in **(A)**. Results shown as mean ± SEM (^∗∗∗∗^*P* < 0.0001). Serum levels of inflammatory cytokines TNF-α **(C)** and IL-1β **(D)** measured after the course of treatment. Results represent mean ± SEM values (^∗∗^*P* < 0.01, ^∗^*P* < 0.05). Figure adapted from Segura-Ibarra et al. (2017), reproduced with permission courtesy of Elsevier.

Aberrant activation of the mammalian target of rapamycin (mTOR) plays an important role in diseases such as cancer (Blanco et al., 2014), leading to the therapeutic exploration of mTOR inhibitors such as RAP. mTOR is also a key player in PAH progression due to its effects on PASMC growth and survival (Goncharova, 2013). Rapamycin has been shown to prevent PAH progression pre-clinically (Houssaini et al., 2013) while clinical exploration of everolimus, a rapalog, led to improvements in pulmonary vascular resistance and 6MWD (Seyfarth et al., 2013). Similar to the aforementioned imatinib, resistance to RAP is a limitation of the drug, stemming from mutations in mTOR or mutations in downstream effectors of mTOR (S6K1 or 4E-BP1) (Huang and Houghton, 2001). Our laboratory recently examined the potential of RAP NPs for the treatment of PAH (Segura-Ibarra et al., 2017). RAP was encapsulated within PEG-PCL polymer micelles measuring ∼17 nm in diameter. In an MCT-induced rat model of PAH, RAP NPs led to a significant increase in RAP in diseased lungs compared to healthy lungs. Similarly, RAP NPs led to an increase in RAP in diseased lungs compared to a free drug formulation. Moreover, NPs were localized primarily in pulmonary vasculature. Following twice a week administration of RAP NPs at the time of PAH induction for a duration of 4 weeks, RAP NPs significantly reduced pulmonary arteriole hypertrophy (**Figures 5A,B**) and RV ventricular remodeling compared to vehicle controls, and prevented increases in right ventricular systolic pressures and phosphorylation of S6, a downstream effector of mTOR. Importantly, compared to a free drug formulation of RAP, a 10% decrease in weight loss associated with RAP was observed in rats receiving RAP NPs, accompanied as well by a decrease (∼50%) in levels of pro-inflammatory cytokines (**Figures 5C,D**).

### NP Delivery of Genetic Material in PAH

Enhanced insights into molecular machinery driving PAH progression, including those involved in inflammation, have resulted in the identification of several viable therapeutic targets, with NP-based delivery platforms enabling gene therapy. As an example, NF-κB is a transcription factor that regulates numerous inflammatory cytokines, including IL-6 and TNF-α, which are involved in PAH (Hoesel and Schmid, 2013). Kimura et al. (2009) examined the role of NF-κB in PAH, as well as the potential for NP-based therapeutics targeting NF-κB as a treatment strategy. An NF-κB decoy oligodeoxynucleotide meant to inhibit binding of NF-κB to the promoter region was encapsulated within poly(ethylene glycol)-*block*-lactide/glycolide (PEG-PLGA) polymer micelles. Resulting NPs measured 44 nm in diameter, and displayed release of ∼40% of NF-κB decoy over 24 h and sustained release over the course of 28 days. Efficacy of NF-κB NPs were examined in preventive (NP intratracheal administration at the time of model induction) and treatment (NP intratracheal administration 21 days after model induction) protocols in an MCT-induced model of PAH. Using FITC-labeled NF-κB for visualization of NPs, FITC signal was found in small arteries, arterioles, small bronchi, and alveoli of diseased lungs at timepoints of 7 and 14 days after administration. In the preventive study, NF-κB positive cells were significantly reduced (> 50%) compared to free NF-κB decoy controls 7 days after model induction. In the treatment study, NF-κB NPs resulted in a significant decrease in RV systolic pressure, RV hypertrophy, and percentage of muscularized pulmonary arteries compared to PBS controls. Moreover, mRNA levels of inflammatory factors such as monocyte chemoattractant protein (MCP) 1, TNF-α, IL-6, and ICAM-1, were reduced by more than 50% following treatment with NF-κB NPs compared to free NF-κB decoy controls, and animal survival rate was increased compared to vehicle controls.

**FIGURE 6:**
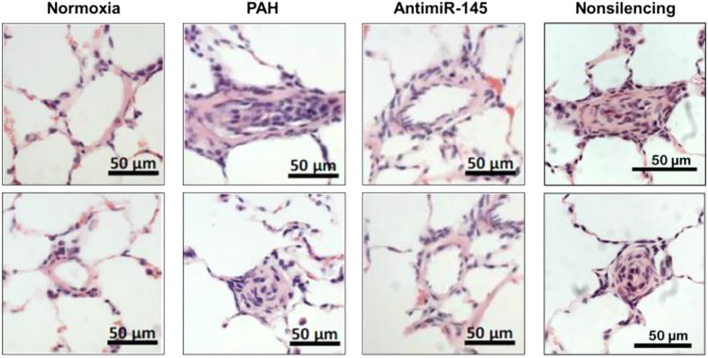
Effects of anti-miR-145 loaded liposomes on arteriole hyperplasia in an MCT-induced model of PAH. Hematoxylin and Eosin (H&E) stained histological sections depicting pulmonary arterioles following anti-miR-145 liposome treatment of rats with Sugen/Hypoxia induced PAH. Results highlight anti-miR-145 liposome treatment compared to controls consisting of healthy controls (normoxia), rats undergoing PAH (PAH), and liposomes containing non-silencing control oligonucleotide (non-silencing). Scale bar represents 50 μm. Figure adapted from McLendon et al. (2015), reproduced with permission courtesy of Elsevier.

In a study by McLendon et al. (2015), NPs were used to deliver anti-sense oligonucleotide against microRNA-145 (miR-145) in hopes of exploiting RNA interference (RNAi) as a viable treatment strategy in PAH. Increased expression of miR-145 has been shown in lungs undergoing PAH, playing a vital role in vascular remodeling and pulmonary artery muscularization (Zhou et al., 2015). Moreover, downregulation of miR-145 prevents the onset of PAH in preclinical models (Caruso et al., 2012). Anti-miR-145 oligonucleotides were encapsulated within cationic lipid nanoconstructs in the range of 80–100 nm in size. Efficacy was examined in a Sugen/Hypoxia model of PAH in rats, wherein NPs were administered IV every 2 weeks starting on week 8 after model induction. Liposomes delivered anti-miR-145 to diseased lungs, and decreased the expression of miR-145 by more than 50%. The median wall thickness of pulmonary arteries was reduced following treatment with anti-miR-145 liposomes (**Figure 6**), with results suggesting that the therapy was capable of repairing vascular remodeling. Moreover, RV systolic pressure decreased by ∼25% and RV hypertrophy was reduced following treatment with anti-miR-145 liposomes compared to non-silencing oligonucleotide controls.

## Conclusion

Pulmonary arterial hypertension results in considerable patient morbidity, proving irreversible and fatal. Present-day pharmacotherapies suffer from considerable limitations. Short-term drug pharmacokinetics, where half-lives are on the order of minutes, contribute to low bioavailability in diseased tissues and adverse side effects. Nanoplatforms have improved the pharmacokinetic profiles of chemotherapeutics, with increased accumulation of NPs in tumors through the EPR effect. Importantly, enhanced accumulation and persistence of NPs has been observed in lungs undergoing PAH following both intravenous and inhalational routes of delivery. Endothelial dysfunction present in diseased lung vasculature results in NP accumulation in pulmonary arterioles, and NPs are found largely associated with vascular cells such as PAECs and PASMCs. Given the vital role these cells play in PAH progression, NPs stand to significantly impact PAH treatment strategies and patient outcomes.

Herein, we have provided an overview of NP-based drug delivery strategies in PAH, with particular emphasis on improvements in vascular remodeling and hemodynamics. Several nanotherapies involved the use of clinically approved drugs for PAH, while others exploited novel signaling pathways and molecular targets. The future of NP-based drug delivery in PAH will surely involve advancements on two fronts. On the one hand, innovations in materials science will lead to sophisticated nanotechnology platforms highly capable of delivering drugs to target cells in diseased lungs. These nanoconstructs will incorporate moieties for successful navigation of barriers to transport to the lungs, facilitate sustained delivery of therapeutics over time, and enable combined delivery of drugs and genetic material for synergistic treatment. Additionally, nanotherapies in PAH will benefit from enhanced understanding of molecular drivers of the disease. Insights into processes of PAH pathogenesis can potentially provide overexpressed surface receptors for active targeting to target cells and provide novel targets for gene therapy. Thus, rational design of NPs that can effectively target diseased lungs combined with molecular-targeted therapeutics will lead to more efficacious treatment outcomes in PAH.

## Author Contributions

All authors listed have made a substantial, direct and intellectual contribution to the work, and approved it for publication.

## Conflict of Interest Statement

The authors declare that the research was conducted in the absence of any commercial or financial relationships that could be construed as a potential conflict of interest.
